# An Exploration of the Application of Step Counter-Based Physical Activity Promotion Programs in Patients With Chronic Obstructive Pulmonary Disease: A Systematic Review

**DOI:** 10.3389/fpubh.2021.691554

**Published:** 2021-09-23

**Authors:** Xiaoyu Han, Peijun Li, Yahui Yang, Xiaodan Liu, Jun Xia, Weibing Wu

**Affiliations:** ^1^Department of Sports Rehabilitation, Shanghai University of Sport, Shanghai, China; ^2^School of Rehabilitation Science, Shanghai University of Traditional Chinese Medicine, Shanghai, China

**Keywords:** chronic obstructive pulmonary disease, physical activity, step counter, application, effect

## Abstract

**Objective:** This paper aimed to systematically review the application methods and components of step counter-based physical activity (PA) promotion programs in patients with chronic obstructive pulmonary disease (COPD). The effects of longer-duration (≥12 weeks) programs on PA, exercise capacity, quality of life, and dyspnea were discussed.

**Methods:** This review was performed in accordance with the preferred reporting items for systematic reviews and meta-analysis. Online data resources PubMed, Web of Science, Embase, and EBSCO were searched. The publication year was limited between January 2000 to August 2020. All randomized controlled trials with ≥12-week duration of step counter-based PA promotion programs of COPD were included. Two researchers independently assessed the quality of the included studies and extracted their characteristics.

**Results:** Nine studies involving 1,450 participants were included. Step counters, counseling, exercise goals, diaries, and tele-communicational approaches were common components of these programs. The PA feedback tools were mostly pedometers (*n* = 8), whereas accelerometers were often used as assessment tools of PA (*n* = 5). All studies implemented counseling: five applied behavioral change theories, and three reported motivational interview techniques simultaneously. Six studies reported detailed exercise goals. The usual exercise goal was to reach a total of 8,000–10,000 steps/day. Three research studies used diaries, and five applied tele-communication approaches to deliver interventions. The programs could be implemented alone (*n* = 4), in combination with exercise training (*n* = 2), or with pulmonary rehabilitation (*n* = 2). All studies showed a significant increase in the PA (≥793 steps/day). Three studies observed a significant improvement in exercise capacity (≥13.4 m), and two reported a significant increase in the quality of life (*p* < 0.05). No study showed significant between-group differences in dyspnea.

**Conclusion:** There are a few studies assessing the impact of long-duration (≥12 weeks) step counter-based interventions in COPD, with different methodologies, although all studies included counseling and exercise goal setting. These interventions seem to have a positive effect on PA. A few studies also showed benefit on exercise capacity and quality of life.

## Introduction

Chronic obstructive pulmonary disease (COPD) is a kind of treatable and preventable lung disease, featuring persistent and progressive airflow limitation (1). In 2015, 3.2 million people died from COPD globally, which was an increase of 11.6% compared with that in 1990 (2). Given dyspnea, fatigue, and exercise intolerance when performing physical activities (PAs), physical inactivity is prevalent among patients with COPD (3). Compared with healthy controls, the steps per day of COPD patients decreased significantly (9,372 ± 3,574 min vs. 3,584 ± 3,360 min, *p* < 0.0001) (4), and were lower than the recommended 5000 steps/day (5); the time spent in activities with mild (160 ± 89 min vs. 80 ± 69 min, *p* = 0.004), moderate (65 ± 70 min vs. 24 ± 29 min, *p* < 0.0001), and high (7 ± 9 min vs. 2 ± 5 min, *p* = 0.01) intensity significantly decreased (5); the time spent sitting (306 ± 108 min vs. 374 ± 139 min, *p* = 0.04) and lying down (29 ± 33 min vs. 87 ± 97 min, *p* = 0.004) significantly increased (6). With the progression of the disease, the PA of patients with COPD was further compromised (7). The PA of COPD was significantly lower than those of rheumatoid arthritis and diabetes based on the guidelines (84, 74, and 72%, respectively*; p* < 0.01) (8, 9). The physical inactivity of patients with COPD was associated with poor health outcomes and is a predictor of a high hospital admission rate (10). A low PA level of COPD is significantly related to reduced pulmonary function and poor quality of life (QoL) (11). Furthermore, objectively measured PA levels were the strongest predictor of all-cause mortality in patients with COPD (12).

Conventional pulmonary rehabilitation (PR) can significantly improve limb muscle strength, exercise capacity, pulmonary symptoms, and health state (13), but it cannot translate these changes to the PA improvement (14, 15). Therefore, a more promising way to promote the PA of patients with COPD is needed. Step counter-based PA programs have been applied to improve the PA of patients with COPD (16). As a kind of objective PA monitoring device, step counters are tools to record PA and to promote behavioral changes (17). Studies have shown that long-duration interventions using PA trackers (≥12 weeks) have great effects on the PA of the elderly (18). However, the employment of components of PA promotion programs in COPD and their effect have not reached an agreement. Therefore, we systematically reviewed the PA program components and application methods of long-term (≥12 weeks) step counter-based PA programs to patients with COPD. The effects of these programs on PA, exercise capacity, QoL, and dyspnea were also explored.

## Research Methods

This study was reported in accordance with the Preferred Reporting Items for Systematic Reviews and Meta-Analysis (19).

### Search Strategies

Online databases PubMed, Web of Science, EBSCO, and Embase were searched to identify relevant studies. The search strategies used for the different databases varied. With PubMed as an example, the research terms were {[(Pulmonary disease, chronic obstructive [MeSH Terms]) OR (COPD) OR (Chronic Obstructive Pulmonary Disease) OR (COAD) OR (Chronic Obstructive Airway Disease) OR (Chronic Obstructive Lung Disease) OR (Airflow Obstruction, Chronic) OR (Airflow Obstructions, Chronic) OR (Chronic Airflow Obstructions) OR (Chronic Airflow Obstruction)] AND {[Exercise (MeSH Terms)] OR (Physical Activity) OR (Physical Exercise) OR (Aerobic Exercise) OR (Aerobic Exercises) OR (Exercise Training) OR (Physical Activities) OR (Activities, Physical) OR (Activity, Physical) OR (Physical Activities) OR (Exercise, Physical) OR (Exercises, Physical) OR (Physical Exercise) OR (Physical Exercises) OR (Acute Exercise) OR (Acute Exercises) OR (Exercise, Acute) OR (Exercises, Acute) OR (Exercise, Isometric) OR (Exercises, Isometric) OR (Isometric Exercises) OR (Isometric Exercise) OR (Exercise, Aerobic) OR (Exercises, Aerobic) OR (Exercise Training) OR (Exercise Trainings)} AND [(motion sensor) OR (physical activity monitor) OR (motion tracker) OR (step counter) OR (pedometer) OR (accelerometer) OR (accelerometry)]}. In addition, possible references and meta-analyses were screened to identify potential studies.

### Inclusion and Exclusion Criteria

The inclusion criteria were as follows: (1) patients diagnosed with COPD based on spirometry (forced expiratory volume in 1 sec/forced vital capacity < 0.7) who had no history of exacerbation within the previous 4 weeks at least; (2) experimental groups (EGs) that received step counter-based PA promotion interventions and control groups (CGs) that received step counters with no instructions or did not receive step counters; (3) outcomes that measured PA (e.g., steps/day, time spent walking, and moderate-to-vigorous PA); (4) duration ≥12 weeks; and (5) randomized controlled trials.

The exclusion criteria were as follows: (1) participants with other chronic diseases; (2) non-wearable step counters; (3) studies that aimed to test feasibility rather than effectiveness; and (4) the absence of PA-related outcome measures.

### Study Collection and Data Extraction

Two researchers independently investigated the potential studies. Abstracts and titles were screened. Full texts were reviewed to identify eligible studies in accordance with the inclusion and exclusion criteria. If a disagreement ensued, then a discussion was initiated with a third researcher to reach a decision. Two researchers extracted basic information (authors and publication year), subject characteristics (sex and pulmonary function), and intervention characteristics (duration, components of intervention, and outcomes) from the studies. Relevant outcomes values were also extracted.

### Quality Assessment

Physiotherapy evidence database scale was used to assess the quality of the included studies (20). A total of 11 items were included in this scale, and 10 was the maximum number of points. The item “eligibility criteria” was not used to calculate scores. Every item should be answered with “yes” (scored 1), “no” (scored 0), or “not clear” (scored 0). Points totaling 9–10, 6–8, 4–5, and <4 indicated excellent, good, moderate, and low study quality, respectively.

## Results

### Study Search and Data Extraction

**Figure 1:**
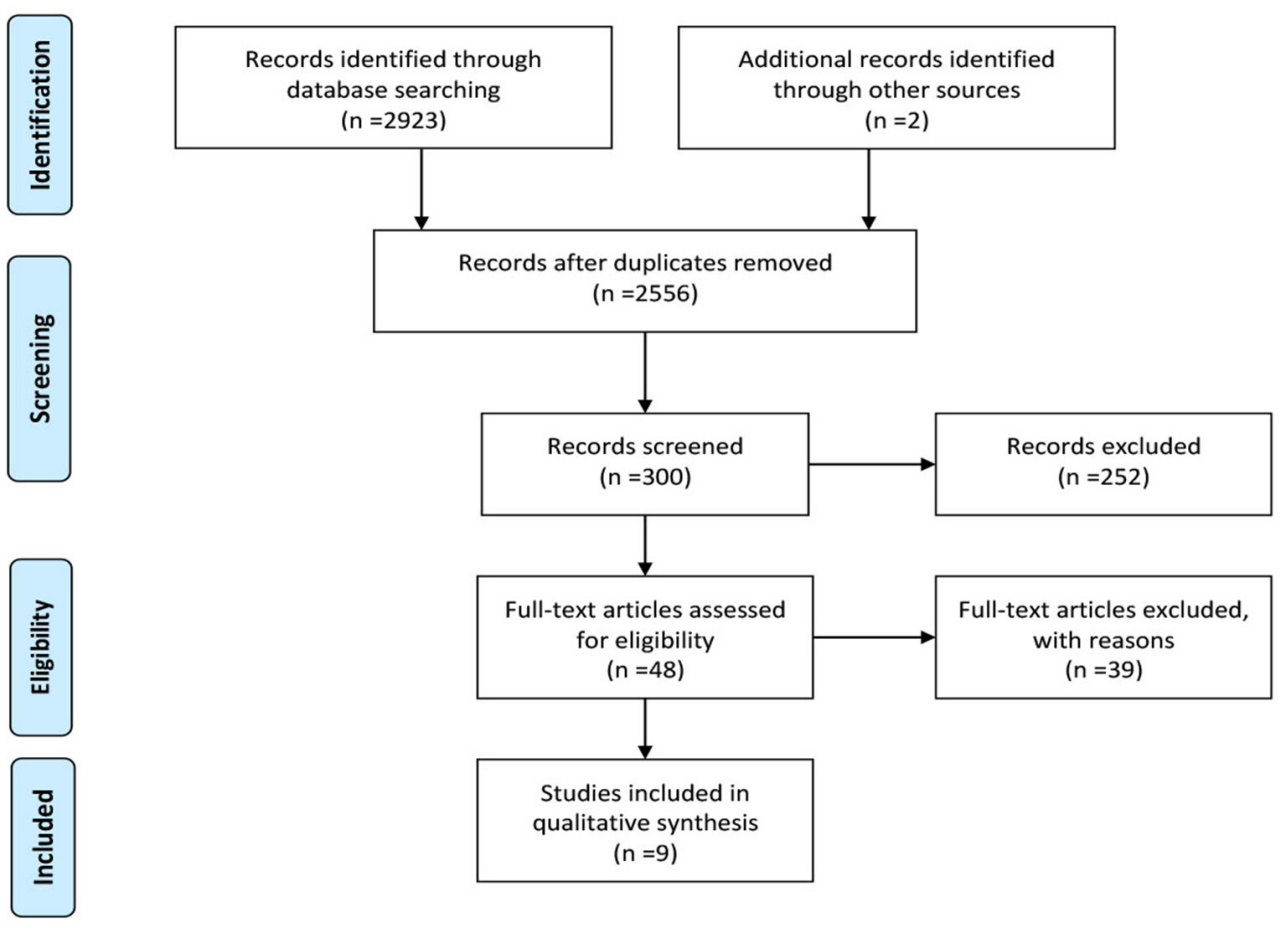
Flow chart.

A total of 2,923 studies were identified as potential studies, with 2 identified through the bibliography. A total of 369 duplicates were removed. After the screening of titles, 300 studies were screened by reading the abstracts, and 48 were retained for eligibility assessment. Finally, nine studies were included (Figure 1). Table 1 shows the results of the extraction of study characteristic.

**Table 1:** Studies about step counter-based PA promotion programs in patients with COPD.

**References**	***n* (male %)^**a**^**	**FEV_**1**_ (%pred)^**a**^**	**Duration**	**Step counters used in PA feedback/assessment**	**CG**	**EG**					**Outcomes**
						**Counseling**	**Goal setting**	**Diary**	**Tele-health**	**Social support**	
Hospes et al. (21)	17 (64.7)/18 (55.6)	61.8 ± 14.4/67.4 ± 17.5	12 weeks	Pedometers/ pedometers	Usual care	Based on behavior change theories, MI	Used	Not used	Not used	Not used	Steps/day↑^c^, leg strength↑^c^, arm strength↑^c^, grip force, 6MWD, SGRQ total↓^c^, SF-36, depression, self-efficacy, motivation↑^c^
Mendoza et al. (22)	50 (66.0)/52 (55.7)	66.0 ± 20.8/66.1 ± 18.2	3 months	Pedometers/pedometers	PA promotion advice, diary	General counseling	Used	Used	Not used	Not used	Steps/day↑^c^, SGRQ total↓^c^, CAT↓^c^, 6MWD↑^c^, mMRC
Altenburg et al. (23)	155 (65.8)^b^	60 (40, 75)^b^	12 weeks	Pedometers/accelerometers	Usual care (participants from PR centers underwent PR)	Based on behavior change theories, MI	Used	Used	Not used	Not used	Steps/day↑^c^, daily PA↑^c^, 6MWD↑^c^ (secondary care group), SF-36, CCQ, CRQ total↑^c^ (secondary care group)
Kawagoshi et al. (24)	15 (93.3)/12 (83.3)	60.6 ± 20.8/58.0 ± 23.2	12 months	Pedometers/accelerometers	Same PR as EG	General counseling	Used	Not used	Not used	Not used	Time spent walking↑^c^, ^d^, ^e^, QF↑^d^, 6MWD↑^d^, ^e^, MRC↓^d^, CRQ total↑^d^, ^e^ (dyspnea↑^d^, fatigue, emotional function, mastery)
Moy et al. (25)	84 (91.7)/154 (84.8)	No data	4 months	Pedometers/pedometers	Wear pedometer but have no related knowledge	Based on behavior change theories	Used	Not used	used	Used	Steps/day↑^c^, ^d^, SGRQ total↓^d^ (symptoms↓^c^, ^d^ activities, impact↓^c^, ^d^)
Cruz et al. (26)	16 (87.5)/16 (81.2)	68.4 ± 19.7/65.5 ± 21.1	3 months (3-month PR+PA promotion plus 3-month PA promotion for EG)	Pedometers/accelerometers	Same PR as EG	Based on behavior change theories	Used	Used	Used	Not used	Steps/day↑^c^, time in total PA↑^c^, time in recommended MVPA↑^c^, time in MVPA↑^c^, 6MWD↑^d^, ^e^, QF↑^d^, ^e^, SGRQ total↓^d^, ^e^ (symptoms, activities↓^d^, ^e^, impact↓^d^, ^e^), self-efficacy
Demeyer et al. (27)	172 (63)/171 (65)	57 ± 21/55 ± 20	12 weeks	Pedometers/accelerometers	PA education	General counseling	Used	Not used	Used	Not used	Steps/day↑^c^, time in MPA↑^c^, walking time↑^d^↓^e^, 6MWD↑^c^, QF, CAT↑^e^, CCQ (metal state, functional state↓^c^, symptoms↑^d^, ^e^), mMRC
Wan et al. (28)	52 (98.1)/57 (98.3)	65.2 ± 21.9/60.2 ± 21.2	3 months	Pedometers/pedometers	Wear pedometer but have no related knowledge	General counseling	Used	Not used	used	Used	Steps/day↑^c^, 6MWD, SGRQ, mMRC↑^d^, motivation to exercise daily↑^d^, COPD knowledge score↑^d^, exercise self-regulatory efficacy↓^d^
Arbillaga-Etxarri et al. (29)	205 (86)/202 (84)	57 ± 18/56 ± 17	12 months	Accelerometers/accelerometers	Same general health counseling and PA recommendation as EG	Based on behavior change theories, MI	Used	Not used	Used	Used	Steps/day↑^c^ (per-protocol analysis set), CCQ, CAT, 6MWD, C-PPAC, HAD

*Data are presented as mean ± SD, n (%) or M (P_25_, P_75_). 6 MWD, 6-min walking distance; CAT, COPD assessment test; CCQ, Clinical COPD Questionnaire; CG, control group; C-PPAC, Clinical visit-PROactive Physical Activity in COPD; CRQ, chronic respiratory questionnaire; EG, experimental group; FEV_1_, forced expiratory volume in 1sec; HAD, hospital anxiety and depression scale; mMRC, modified Medical Research Council; MI, motivational interview; MPA, at least moderate intense physical activity; MRC, Medical Research Council; MVPA, moderate-to-vigorous physical activity; PA, physical activity; PR, pulmonary rehabilitation; QF, quadriceps femoris muscle force; SGRQ, St. George's Respiratory Questionnaire; SF-36, Short Form 36. ↑ Data increased significantly. ↓ Data decreased significantly.*

a *CG/EG.*

b *Data of both groups.*

c *Between groups.*

d *Within EG.*

e *Within CG*.

### Quality of Included Studies

Table 2 presents the results of quality assessment. No low-quality study was detected. Four and five studies were of moderate and good quality, respectively. All studies were comparable in terms of baseline characteristics between groups, reported between-group difference, and point measure. Four studies reported allocation concealment, and four reported measures of key outcomes for >85% of the participants. Three studies performed intention-to-treat analysis. Four studies reported assessor blinding. Two studies implemented participant blinding, and no research reported therapist blinding.

**Table 2:** Quality assessment (20).

**References**	**Eligibility criteria**	**Random allocation**	**Concealed allocation**	**Baseline similarity**	**Participants blinding**	**Therapists blinding**	**Assessor blinding**	**Measures for >85%**	**ITT**	**Between-group difference**	**Point measure and variability**	**Total score**
Hospes et al. (21)	Reported	Y	N	Y	N	N	N	Y	N	Y	Y	5
Mendoza et al. (22)	Reported	Y	N	Y	N	N	Y	Y	Y	Y	Y	7
Altenburg et al. (23)	Reported	Y	N	Y	N	N	N	N	N	Y	Y	4
Kawagoshi et al. (24)	Reported	Y	N	Y	N	N	N	N	N	Y	Y	4
Moy et al. (25)	Reported	Y	N	Y	N	N	N	Y	N	Y	Y	5
Cruz et al. (26)	Reported	Y	Y	Y	Y	N	N	N	N	Y	Y	6
Demeyer et al. (27)	Reported	Y	Y	Y	N	N	N	N	Y	Y	Y	6
Wan et al. (28)	Reported	Y	Y	Y	N	N	Y	Y	N	Y	Y	7
Arbillaga-Etxarri et al. (29)	Reported	Y	Y	Y	Y	N	Y	N	Y	Y	Y	8

*ITT, intention-to-treat analysis*.

### Characteristics of Studies

Step counter-based PA promotion programs are comprehensive intervention techniques that included the following components: step counters, counseling, goal setting, diaries, and social support. The main procedures of the programs are as follows.

**Figure 2:**
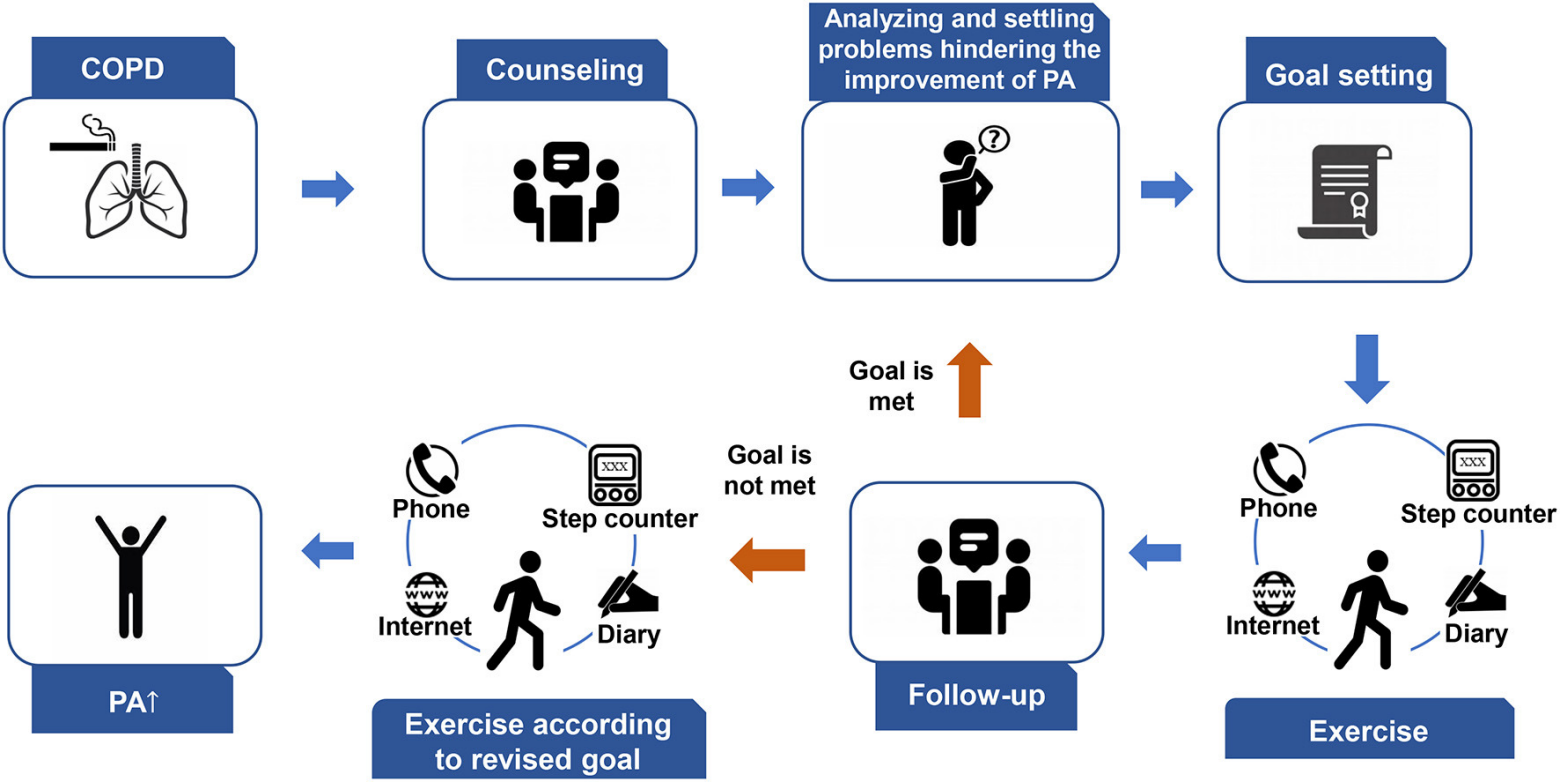
Procedure of step counter-based PA promotion programs of patients with COPD. COPD, chronic obstructive pulmonary disease; PA, physical activity.

(i) Counseling: Researcher-led counseling principally aimed to inform patients of the relationship between PA and COPD, strengthen motivation to improve PA, and remove PA promotion obstacles. The PA data during a specific period were shown to researchers in counseling sessions, which was a kind of feedback. Several counseling sessions should be performed before and during the intervention. All the studies included counseling. Counseling could be implemented as behavioral change theory-based interviews (21, 23, 25, 26, 29), motivational interviews (MIs) (21, 23, 29), or general counseling (22, 24, 27, 28). Behavioral change theories are theoretical frameworks that are built based on psychological and behavioral knowledge and can effectively explain the process of behavior occurrence, change, and maintenance. The theoretical frameworks used in these programs were self-regulation theory (25), self-determination theory (21), principles of goal-setting and implementation (21, 23, 24), transtheoretical modeling (21, 29), social cognitive theory (26), and the relapse prevention model (21). MI was developed by William et al. to settle risky alcohol intake and then evolved into client-centered counseling aimed at changing behavior by increasing the intrinsic motivation (30).
(ii) Exercise goal setting: Exercise goals were usually formulated based on the PA data, and they were presented as average steps per day (22, 24–28). One study instructed participants to walk at least one validated trail per day at least 5 days per week, and at a pace reaching Borg scale 4–6 (29). Goals were mostly revised periodically and individually. Seven studies reported detailed exercise goals (22, 24–29).
(iii) Instructing patients to comprehensively use step counters, diaries, and social support to promote PA. Tele-communication approaches were used to deliver interventions. Step-count displayed on the step counters provided participants PA feedback (25, 27, 28). In a study (24), investigators collected PA data and revised it during the counseling approaches. This was also an approach to provide participants PA feedback. Patients should follow the instruction for wearing step counters in their daily life. Eight studies (21–28) used pedometers as feedback tools during the PA promotion process, whereas one study (29) used accelerometers. Four (21, 22, 25, 28) and five studies (23, 24, 26, 27, 29) used pedometers and accelerometers as PA assessment tools, respectively. In PA data collection and promotion, participants were encouraged to wear step counters from the moment they stood up until they went to bed, except when bathing or swimming. Most studies (25–29) defined ≥8 h a day and ≥2 days a week as valid periods for wearing step counters, as validated by Demeyer et al. (31). Moy et al. (25) and Wan et al. (28) added ≥100 steps/day as an extra condition. Diaries were subjective feedback of PA and could strengthen self-monitoring. Participants in three studies were asked to record their steps/day and time when performing PAs (22, 23, 26). Social support enabled COPD patients to participate in PAs (32, 33). Three studies implemented social support (25, 28, 29). Two of them (25, 28) conducted online forums, and one (29) organized walking groups. Meanwhile, researchers could strengthen the motivation and confidence of patients to complete the program through phone calls, text messages, and the Internet.
(iv) Follow-up: Revisits were needed and often performed as a counseling approach. They were conducted to collect PA data, settle the problems hindering PA's improvement of PA, and revise the step-count goals. Figure 2 shows the procedure of step counter-based PA promotion programs for patients with COPD.

The programs could be implemented alone (21, 22, 25, 28), in combination with exercise training (27, 29) or with PR (24, 26). Two studies implemented usual care in CGs (21, 23). Given the diversity of participant recruitment settings in one research (23), participants in the CG received different usual care to maintain the previous therapy. Two studies launched the same PR as EGs in CGs (24, 26). CGs of three studies received PA promotion education (22, 27, 29). Two studies gave pedometers to CGs, but PA-related knowledge was not proposed (25, 28).

### Outcome Measures of Step Counter-Based PA Promotion Programs in Patients With COPD

#### PA

Eight studies (21–23, 25–29) assessed steps/day and showed significant improvement (≥793 steps/day). Demeyer et al. (27) reported a significant increase in moderate-intensity PA in the EG compared with the CG of 10.4, 95%CI (6.1–14.7) min/day. Altenburg et al. (23) investigated the significant increase in the daily PA. Cruz et al. (26) confirmed the significant improvement between groups in terms of the total PA time (20.3 ± 24.2 min/day vs. 3.8 ± 7.4 min/day, *p* = 0.033), time for recommended moderate-to-vigorous physical activity (MVPA) (23.3 ± 28.6 min/day vs. 4.3 ± 7.3 min/day, *p* = 0.036), and time in MVPA (57.8 ± 32.8 min/ day vs. 26.7 ± 19.6 min/day, *p* = 0.007). Kawagoshi et al. (24) reported that changes in walking time in the EG significantly increased compared with the CG (51.3 ± 63.7 min/day vs. 12.3 ± 25.5 min/day, *p* = 0.036).

#### Exercise Capacity

Eight studies (21–24, 26–29) evaluated the exercise capacity by 6 min walking distance. Two of these works showed significant increases between groups [0.81 (−7.7 to 6.1), *p* = 0.009 and −0.7 ± 24.4 m vs. 12.4 ± 34.6 m, *p* = 0.03, respectively] (22, 27). One research (23) reported a significant between-group increase in the exercise capacity of a subgroup of participants recruited from secondary care [23 (0–50.9) m vs. 3.5 (−32.2–26.8) m, *p* = 0.049]. Two studies (24, 26) reported a significant increase within two groups (EG = 445 ± 138 m vs. 369 ± 119 m, CG = 467 ± 151 m vs. 404 ± 148 m and EG = 547.9 ± 47.9 m vs. 493.8 ± 63.0 m, CG = 529.7 ± 57.2 m vs. 476.2 ± 54.9 m, respectively), but no between-group difference was observed. No effect on exercise capacity was observed in three studies (21, 28, 29).

#### Dyspnea

Three studies (22, 27, 28) evaluated dyspnea by modified Medical Research Council dyspnea scale (mMRC), and one (24) used the Medical Research Council dyspnea scale. No significant improvement was found between groups.

#### QoL

All the studies assessed the QoL. St. George's respiratory questionnaire (SGRQ) (21, 22, 25, 26, 28), COPD clinical questionnaire (CCQ) (23, 27, 29), chronic respiratory questionnaire (CRQ) (23, 24), COPD assessment test (CAT) (22, 27, 29), and Short form-36 (23) were used to evaluate the QoL. Two studies (21, 22) observed a significant increase in QoL total score (*p* = 0.05 and *p* = 0.02, respectively). Within-group difference was found in two studies (24, 26) (*EG* = 98 ± 20 vs. 108 ± 19, *CG* = 99 ± 19 vs. 110 ± 19 for CRQ scores and *EG* = 31.5 ± 15.7 vs. 24.0 ± 13.6, *CG* = 34.9 ± 14.7 vs. 26.0 ± 15.2 for SGRQ scores, respectively). The domains of QoL questionnaire improved significantly between groups in two studies [−4.6 (−9.0 to −0.1) for the symptom domain of SGRQ, −3.3 (−6.7 to −0.2) for the impact domain of SGRQ; −0.203 (−0.382 to −0.024) for functional state domain of CCQ] (25, 27). No change in QoL was found in two studies (28, 29).

## Discussion

Step counter-based PA promotion programs are approaches to be used in COPD. However, no consensus has been reached regarding the application methods of its components, and their effects are also unclear. Hence, we reviewed the current application of step counter-based PA promotion programs in COPD to summarize their components and to detect their effects. Potential methods to improve the effect were proposed.

### Components of Step Counter-Based PA Promotion Programs in Patients With COPD

Step counters are cheap, accessible, and wearable devices. They show steps/day, walking distance, and energy expenditure and are widely used to promote and monitor PA (10, 11, 17). Step counters include pedometers and accelerometers. Bi-axial and tri-axial accelerometers are more sensitive in detecting PA (34) compared with pedometers. The validity of accelerometers in the measurement of PA of COPD patients in daily life and in an experimental environment has been confirmed previously (35, 36). During PA promotion, step counters were used to obtain feedback and to monitor the PA of COPD patients. Regarding their effects on PA, no difference was found between the use of pedometers and accelerometers as assessment tools. This finding was consistent with those obtained in previous studies (16). Eight studies (21–23, 25–29) applied pedometers as objective feedback tool, and one study (24) used accelerometers. Thus, the analysis of the difference between pedometers and accelerometers when applied as PA feedback tool is difficult. However, Cooper et al. (37) observed that accelerometers alone or combined with other components were more effective than pedometers. They attributed this result to the poor accuracy of pedometers when used to measure the low-speed PA of the elderly. Pedometers are less accurate in assessing low-speed PAs. In PA promotion, they were mostly used as feedback tools to ensure that participants were aware of their PA and achieved their goals. Moreover, the underestimated PA obtained with pedometers may motivate participants to walk more, thereby increasing their PA.

Counseling, goal-setting, and tele-communication are also components of step counter-based PA promotion programs for COPD. Topics of counseling include benefits from PA, solving problems that hinder the improvement of PA, and increasing or maintaining motivation to improve PA. Behavioral change theory-based counseling is more effective than counseling approaches that did not apply such theory (38). MI techniques are common in behavioral change counseling. The professionalism of presiders has a great relationship with the effect of counseling, but few studies (21, 23, 29) reported presiders who were trained in the use of MI techniques. Although the MIs delivered by any presider type offer positive outcomes, mental health presiders and multidisciplinary teams can reach a statistical significance (30). Exercise goals are usually set and revised during counseling in the form of steps/day. Exercise goals are predictors of PA improvement (17). Studies showed varied goals, the long-term goal was approximately 8,000–10,000 steps/day. Exercise intensity is important in the World Health Organization recommendation of PA (39). Studies translated intensity into steps/day and demonstrated its practical application (40, 41). However, only one (29) study instructed participants to walk at a speed reaching Borg 4–6. Two studies (26, 27) measured intensity-related outcomes. Future research should focus on a comprehensive approach in goal setting.

Tele-communication approaches, such as phone calls, websites, text messages, and apps, were used to visit, revise goals, instruct exercise training, and conduct PR. This kind of intervention delivery allows easy accessibility to medical resources and saves transportation time and expenditure. Two studies (25, 28) conducted an online forum, and one (29) organized walking groups. These activities aimed to provide social support that could enable COPD patients to participate in PAs (32, 33). Social support was observed from other COPD patients in the included studies. Those with similar medical histories eased their communication and provided positive information to improve PA.

The effect of every single component to promote PA was detected (16, 17, 32, 33, 42), but the influence of their combination is still unclear. PA is a complex behavior, and multifactorial changes (intrapersonal, interpersonal, and physical environmental factors) can improve its promotion (43). Step counters, counseling, goal setting, diaries, and several tele-communication approaches changed the intrapersonal factors hindering PA improvement in COPD patients. Few studies intended to modify interpersonal factors (such as social support). Interventions on other factors that should be settled were not proposed.

### Effect of Step Counter-Based PA Promotion Programs on Patients With COPD

Studies showed that significant increases in the steps/day reached the minimum clinically important difference (MCID), which is 600 steps/day (44). This result is in line with that obtained by Qiu et al. (16), who indicated that step counter-based PA promotion programs could increase the PA of COPD significantly compared with exercise training, long-term oxygen therapy, and neuromuscular electrical stimulation. Armstrong et al. (45) performed a meta-analysis that included minimal duration of 8 weeks and showed that step counter-based PA programs alone and in combination with PR could effectively improve PA. Our study showed the significant effect of these programs on a long duration (≥12 weeks). Mantoani et al. (42) demonstrated that activity monitoring devices effectively promoted the PA of COPD patients; the potential of these devices in promoting the PA of patients with COPD was assumed. Liu et al. (46) believed that compared with the usual care and health education, step counters better promote the PA of elders. Step counters are promising devices that can promote the PA of patients with diabetes and cancer (47–49). In general, step counter-based PA promotion programs may improve the PA of patients with COPD.

Our study showed limited evidence on the effect of step counter-based PA promotion programs on exercise capacity in patients with COPD. Studies demonstrated that the significant increase in exercise capacity did not exceed the MCID (30 m) (50). These programs did not cause a clinically significant improvement in exercise capacity. Most of the included studies set goals of specific steps/day, which can be achieved by walking, which is a low-intensity exercise (51). However, changes in the exercise capacity need regular high-intensity exercises (52). This condition may lead to a limited improvement in exercise capacity. Setting goals about exercise intensity to achieve a dyspnea or a fatigue rating of 4–6 in Borg scale while exercising may improve exercise capacity (27, 29). In the study included intensity in exercise goal, exercise capacity was not improved significantly. It may be due to the lack of supervision, which is necessary to ensure that participants achieved the intensity (29). Qiu et al. (16) also did not demonstrate a clinically significant increase in the exercise capacity after step counter-based PA promotion intervention in patients with COPD.

Dyspnea is a vital cause of physical inactivity (53). Dynamic hyperinflation, breath burden caused by airway stenosis, and respiratory muscle dysfunction are causes of dyspnea (54). Numerous studies assessed dyspnea by using mMRC, but none of them found a significant improvement between groups after intervention. One study (24) reported a significant within-group improvement. This result may be due to the fact that relief of dyspnea could be achieved only when the threshold of exercise-induced dyspnea was reached. However, goals could be reached by low-intensity walking which may not be enough to reach exercise-induced dyspnea. Moreover, the study showed a within-group significance when the exercise intervention was sustained for 12 months, indicating that duration may be related to the effect on dyspnea. Involvement of exercise intensity in the goals and extension of the duration may help alleviate dyspnea.

The improvement of QoL is an important aim of PR. Two studies (21, 22) found significant improvement, but the value exceeded the MCID (4 points) (55). Studies (24, 26) that implemented PR in both of the groups observed significant within-group differences. Significant between-group differences of QoL total scores were not detected in two studies (28, 29). It may be because the baseline QoL levels of the participant in Arbillaga-Etxarri et al. (29) were better compare with another study which also measured with CAT (12 ± 7 vs. 15.5 ± 8.9 points, respectively) (22). Different participants enrollment seasons might lead to the limited effects on QoL in the study of Wan et al. (28).

### Limitations

Long-duration step counter-based PA promotion programs were included to detect their components and to discuss their effects on PA, exercise, dyspnea, and the QoL. Heterogeneity existed because of the varied components (some participants applied behavioral change theories or diaries, whereas others did not) and application methods (PA promotion programs were conducted alone or in combination with other programs). Thus, meta-analysis cannot be performed. This condition limited the quantitative evidence of this study. Subgroup analysis cannot be performed, and thus, the effects of program components on the outcomes were hard to detect. Only studies published in English were included; possibly, relevant research works published in other language were missed. Most studies were launched in Europe and Americas, thereby implying possible regional bias.

## Conclusion

Step counter-based PA promotion programs in patients with COPD consisted of different components, such as step counters, counseling, goal setting, diaries, and social support. All the studies included counseling and goal setting. Behavioral change theories and MI techniques could be applied during counseling. Step-count goals were most commonly used. Step counter-based PA promotion programs in COPD with a duration of ≥12 weeks were promising approaches to promote the PA of patients with COPD and could be used in clinical management.

## Author Contributions

XH, WW, and JX had the idea for the article. XH, PL, and YY performed the literature search and data extraction. WW, JX, and XH drafted the work. WW, JX, XH, PL, YY, and XL contributed to the interpretation of the data and revised the work. All authors have read and approved the final manuscript.

## Funding

This work was funded by the National Natural Science Foundation of China (81902307 and 82072551).

## Conflict of Interest

The authors declare that the research was conducted in the absence of any commercial or financial relationships that could be construed as a potential conflict of interest.

## Publisher's Note

All claims expressed in this article are solely those of the authors and do not necessarily represent those of their affiliated organizations, or those of the publisher, the editors and the reviewers. Any product that may be evaluated in this article, or claim that may be made by its manufacturer, is not guaranteed or endorsed by the publisher.
